# Training for eye contact modulates gaze following in dogs

**DOI:** 10.1016/j.anbehav.2015.04.020

**Published:** 2015-08

**Authors:** Lisa J. Wallis, Friederike Range, Corsin A. Müller, Samuel Serisier, Ludwig Huber, Zsófia Virányi

**Affiliations:** a Clever Dog Lab, Messerli Research Institute, University of Veterinary Medicine Vienna, Medical University of Vienna, University of Vienna, Vienna, Austria; b Department of Cognitive Biology, University of Vienna, Vienna, Austria; c Royal Canin Research Center, Aimargues, France

**Keywords:** ageing, border collie, clicker, development, dog, eye contact, gaze following, habituation, life span, training

## Abstract

Following human gaze in dogs and human infants can be considered a socially facilitated orientation response, which in object choice tasks is modulated by human-given ostensive cues. Despite their similarities to human infants, and extensive skills in reading human cues in foraging contexts, no evidence that dogs follow gaze into distant space has been found. We re-examined this question, and additionally whether dogs' propensity to follow gaze was affected by age and/or training to pay attention to humans. We tested a cross-sectional sample of 145 border collies aged 6 months to 14 years with different amounts of training over their lives. The dogs' gaze-following response in test and control conditions before and after training for initiating eye contact with the experimenter was compared with that of a second group of 13 border collies trained to touch a ball with their paw. Our results provide the first evidence that dogs can follow human gaze into distant space. Although we found no age effect on gaze following, the youngest and oldest age groups were more distractible, which resulted in a higher number of looks in the test and control conditions. Extensive lifelong formal training as well as short-term training for eye contact decreased dogs' tendency to follow gaze and increased their duration of gaze to the face. The reduction in gaze following after training for eye contact cannot be explained by fatigue or short-term habituation, as in the second group gaze following increased after a different training of the same length. Training for eye contact created a competing tendency to fixate the face, which prevented the dogs from following the directional cues. We conclude that following human gaze into distant space in dogs is modulated by training, which may explain why dogs perform poorly in comparison to other species in this task.

## Highlights

• We provide the first evidence that dogs can follow human gaze to distant space.
• There were no age effects on gaze following to human gaze cues.
• Gazing patterns of young and elderly dogs were influenced by higher distractibility.
• Training decreased dogs' gaze following and increased gaze to the human face.
• Dogs' tendency to follow human gaze is modulated by training for eye contact.

In humans, a crucial feature of social life and communication is eye gaze, which plays a central role in social cognition. Gaze following, the ability to monitor and match another's head and eye orientation by following gaze direction into distant space, has been extensively studied in human infants. The first such study by Scaife and Bruner (1975) tested infants (of different ages) seated across from an adult experimenter who addressed the infant before turning to look to the side of the room for a few seconds. This and many subsequent studies indicate that the ability to follow gaze improves as the infant develops. This process is influenced by various factors such as perceptual skills and preferences, habituation, reward-driven learning, social environment and spatial layout (Moore, 2008; Triesch, Teuscher, Deák, & Carlson, 2006).

Although several studies have highlighted the importance of investigating age differences in social cognition, especially in elderly humans, for whom reduced social communication and interaction skills have been found in comparison to middle-aged subjects (Henry, von Hippel, & Baynes, 2009; Slessor, Laird, Phillips, Bull, & Filippou, 2010), there are few life span studies of gaze following. The human literature has focused almost entirely on infants in their first 18 months of life, but also studies testing gaze following in chimpanzees, *Pan troglodytes*, have focused mostly on juvenile or adult animals (Teufel, Gutmann, Pirow, & Fischer, 2010).

Comparative studies in nonhuman animal species can help to shed some light on the evolutionary origins and mechanisms of gaze following (Gómez, 2005). A species of particular interest for comparative studies is the domestic dog, *Canis familiaris*. Dogs share an evolutionary and developmental history with humans as a result of their domestication, and there is ample evidence that dogs have specialized skills in reading human-given cues (Kaminski, 2009). Dogs outperform nonhuman primates in following human gaze in object choice tasks (Cooper et al., 2003; Hare, Brown, Williamson, & Tomasello, 2002), and their gaze following, as is that of preverbal infants, is modulated by ostensive cuing such as direct gaze and addressing by the person, who then indicates with her gaze one of two objects or which of two containers is baited with food (Téglás, Gergely, Kupán, Miklósi, & Topál, 2012).

However, despite the human-like performance of dogs in following human-given cues in object choice tasks, there is conflicting evidence of whether dogs follow human gaze in nonforaging contexts. Recently, Met, Miklósi, and Lakatos (2014) found evidence that some dogs follow gaze to and around a barrier, even in nonforaging situations; however, as a group, dogs performed below chance. Additionally Agnetta, Hare, and Tomasello (2000) found no indication that dogs follow human gaze into distant space.

Since gaze following into distant space has been documented in many species such as apes (Bräuer, Call, & Tomasello, 2005; Povinelli & Eddy, 1997), domesticated goats, *Capra aegagrus hircus* (Kaminski, Riedel, Call, & Tomasello, 2005), several bird species (Kehmeier, Schloegl, Scheiber, & Weiss, 2011; Loretto, Schloegl, & Bugnyar, 2010), the red-footed tortoise, *Chelonoidis carbonaria* (Wilkinson, Mandl, Bugnyar, & Huber, 2010) and wolves, *Canis lupus* (Range & Virányi, 2011), we would expect the gaze-following response to be present also in dogs. So why do we find so little evidence that dogs follow gaze outside of object choice situations?

First, we can hypothesize that as gaze following is likely to be a product of both reflexive and learnt mechanisms (Ricciardelli, Carcagno, Vallar, & Bricolo, 2013), one explanation could be that dogs may lose their reflexive responding to human gaze cues through long-term habituation over an individual's lifetime living with human companions (the long-term habituation hypothesis). Owners often turn and gaze at objects and stimuli that are irrelevant to dogs in their daily lives, which may lead to a gradual loss of the dogs' gaze-following response. Thus we could expect young dogs' gaze-following response to be more automatic and therefore more frequent than in adult dogs, which have been affected more strongly by learnt gaze responses.

Second, dogs' lack of response to human gaze to distant space may be explained by their training. One of the first training exercises recommended for owners when getting a puppy is to condition the dog's name as an orienting cue, and to develop eye contact with the owner (Howell & Bennett, 2011). Dogs receive this training in various forms of formal training, such as in puppy school, and during obedience, agility and trick training. After giving relevant ostensive cues, which encourages the dog to pay attention, the owner then gives the next specific verbal command or visual signal usual for that training context (e.g. ‘Muffin’ and ‘come’). Dogs may pay attention to the whole of the owner's body, hand or face when anticipating the next cue (for example body orientation (used in agility), specific hand signals (used in obedience tasks) and so on). Therefore, the effects of such formal training may increase the dog's frequency and duration of fixations to the owner (while waiting for the next cue typical for the given training context), which may then interfere with the dog's response when humans present directional gaze cues that are not part of the formal training. Hereafter we refer to this explanation as the formal training hypothesis.

On the other hand, in their daily lives dogs are repeatedly asked to look at humans in many different situations in which dogs may need more flexibility in detecting the relevant communicative cues of their human partners. Such informal training for increased attention to humans is, therefore, likely to increase the chances that dogs will be able to detect human cues, such as gaze cues, and thus may increase the likelihood that the dog may follow human gaze. Since dogs have the opportunity to learn about these cues and to generalize them to different contexts over their lives, we refer to this explanation as the lifelong learning hypothesis.

There is experimental evidence that even short-term training can affect dogs' human-directed attention (Bentosela, Barrera, Jakovcevic, Elgier, & Mustaca, 2008; Wallis et al., 2014). Short-term training for initiating eye contact (depending on the details and the context of the training) may have a two-fold effect on gaze following: either facilitating it, as proposed by the lifelong learning hypothesis, or hindering it, according to the formal training hypothesis. To examine how such short-term training affects dogs' readiness to follow human gaze cues, we tested the dogs' gaze-following response twice, before and after training to initiate eye contact with the experimenter. On the one hand this training may serve to increase the dogs' attention to the experimenter and thus may confirm the lifelong learning hypothesis, if we find that after such training, the dogs' gaze-following propensity increases. Or, on the other hand, since our short-term training to initiate eye contact follows a specific sequence of events (dog looks up at the experimenter's face, the experimenter uses a clicker to mark the behaviour and then rewards the dog with food), the effect of this training may support the formal training hypothesis, where we would expect that the dogs would follow gaze less after than before the training.

The aims of this study were to re-examine the question whether dogs are capable of following human gaze into distant space and, if so, to investigate through age effects whether the propensity to follow gaze is affected by long-term habituation to directional gaze cues and/or training to focus their attention on humans. Thus, we tested dogs of different ages that had a shorter or longer time to habituate to human gaze cues or to learn to pay attention to relevant human-given cues. We also addressed the potential effect of formal training by examining the influence of lifelong training of different intensity. Finally, we aimed to experimentally test the effects of formal training and of learning to pay attention to humans, by comparing the gaze-following propensity of the dogs before and after training to initiate eye contact with the experimenter. To examine the effects of fatigue and/or short-term habituation during repeated testing, an additional group of dogs was tested using the same procedure, but without being trained for eye contact (instead they were trained to touch a tennis ball with their paw). Our predictions were that if long-term habituation was a key factor, older dogs would follow the gaze of the experimenter less than younger dogs. If, however, lifelong learning to pay attention to humans was important, older dogs would follow gaze more than younger ones, and also short-term training for initiating eye contact would increase gaze following. And finally, if formal training had an influence, highly trained dogs would follow gaze less than dogs with little training experience, and also short-term training for initiating eye contact would decrease the propensity of the dogs to follow gaze.

## Methods

### Subjects

One hundred and forty-five dogs ranging in age from 6 months to 13 years and 10 months were divided into seven groups according to age (Table 1). All recruited dogs were border collies kept as family pets to exclude effects of different developmental and ageing speeds of different breeds. The age groups were chosen according to the timing of the main life span developmental stages in the border collie (late puppyhood, adolescence, early adulthood, middle age, late adulthood, senior and geriatric, Siegal & Barlough, 1995). Dogs that were reported by the owner (via questionnaire) as suffering from any detrimental behavioural or cognitive effects of old age consistent with a diagnosis of canine cognitive dysfunction were excluded from the sample. Also excluded were dogs that were not medically fit, including dogs with eye abnormalities.

The gaze-following test was part of an extensive two-part cognitive battery (‘Vienna Canine Cognitive Battery’, Wallis et al., n.d.), in which all dogs participated. Dogs had prior experience of working with the experimenter, and had visited the lab on a minimum of three occasions before the gaze-following test.

A lifelong formal training score was calculated for each dog using the results from an extensive demographic questionnaire filled in by the owners during the cognitive battery testing. Thirteen different training types were specified and are listed here from highest to lowest participation: puppy school, agility, basic obedience, dancing/trick training, sheep dog training, high-level obedience, companion dog training, nose work, other (including participation in other tests at the lab), therapy dog, dummy training, search-and-rescue training and protection training. Owners reported their dogs' past and current training attendance, and dogs were scored as follows: no experience = 0, sporadic training = 1, once or twice a month = 2, once or twice a week = 3 and completed training (with or without an exam) = 4. Scores for each individual for each training type were calculated up to a maximum score of 52 points. Dogs participated in an average of five different training types. Since training score was correlated with age (Spearman correlation: *r*_S_ = 0.458, *P* = 0.001), training score and age were analysed separately in all models.

An additional 13 dogs (five females, eight males; average age 48 months; range 11–112 months) were recruited separately in order to test a second group that did not receive training for initiating eye contact. These dogs did not participate in the cognitive battery, but were familiar with the lab and had been tested previously in other studies. Neither the 145 dogs in the main sample nor the additional 13 dogs in the control had been tested previously in gaze-following tasks.

### Ethical Note

This study was discussed and approved by the institutional ethics and animal welfare committee at the University of Veterinary Medicine Vienna in accordance with Good Scientific Practice guidelines and national legislation (http://www.vetmeduni.ac.at/fileadmin/v/z/forschung/GoodScientificPractice_English.pdf). All subjects that participated in the study were family pets, and reward-based training was utilized in all tests conducted, with no potentially harmful experimental manipulations.

### Test Setting and Procedure

The same experimenter (L.W.) conducted all the tests in an experimental room measuring 5 m × 6 m at the Clever Dog Lab. Along one 6 m wall in the test room there were two doors located approximately 2 m apart. The room was empty apart from a small table standing next to the side wall and a chair for the owner.

#### Phase 1

**Figure 1:**
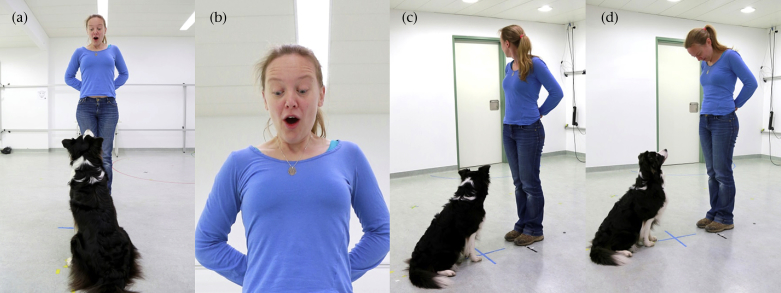
(a) The experimenter centred the dog in the room and gained its attention by calling its name and the command ‘watch’. (b) As soon as the dog looked into her face she immediately made a surprised expression. The gaze cue was then delivered to the dogs in the (c) test and (d) control conditions.

At the beginning of the experiment, the owners entered the experimental room with their dog, released it from the leash, and then sat positioned at the back wall of the experimental room and filled in a questionnaire on an iPad. Owners were instructed to ignore their dog and the actions of the experimenter, and to be as quiet and still as possible. All owners followed these guidelines, and did not attempt to interact with their dogs. The experimenter stood in the centre of the room facing either the windows or the table. She lured the dog into position in the centre of the room sitting in front of her by calling its name and using a small piece of sausage, and then obtained the dog's attention using the ‘watch’ command if necessary. If possible, she held her hands behind her back, but on some occasions it was necessary to point to her face when the dog did not take up eye contact. Looking into the experimenter's eyes in the presence of potential distracters, such as the owner and the food (placed out of reach of the dogs on a table) was the first task dogs needed to fulfil and a precondition of being tested on gaze following. As soon as the dog looked up into her face, the experimenter gave a surprised expression (raised eyebrows, wide eyes, open mouth and intake of breath, see Fig. 1a, b) and either turned her head swiftly and looked to the door for 10 s (test condition Fig. 1c) or looked down at her feet for 10 s (control condition Fig. 1d). The cue was presented for a total of 10 s to enable the recording of the first detectable head turn of the dog away from the experimenter, in line with previous studies on gaze following in mammals (Bräuer et al., 2005; Call, Hare, & Tomasello, 1998; Kaminski et al., 2005; Range & Virányi, 2011). The order of presentation (test/control) was counterbalanced, as was the direction of looking at the door (right/left). In the first session of gaze following two test and two control trials were performed (see the Supplementary Material for a video of the test and control conditions).

#### Phase 2

##### Group eye

After the first gaze-following session, 145 dogs received an intensive training session to initiate eye contact with the experimenter. The experimenter used a secondary reinforcer (clicker) to mark the correct behaviour of looking up into her face, and immediately rewarded the dog after each occasion by throwing onto the floor a small piece of sausage obtained from a food pouch on her back. To initially attract the dog's attention, the experimenter first threw food onto the floor, and then remained motionless waiting for eye contact. No commands were given by the experimenter, and if the dog wandered more than 2 m away from her, she rustled the bag containing the sausage to attract the dog's attention. Importantly, during this training the experimenter never looked to the side; thus the dogs were not trained on gaze following but to look up into her face and establish eye contact. There was no criterion required in the training; each dog participated for a total of 5 min, during which over 95% of the sample achieved a minimum of 20 clicks and rewards.

##### Group ball

The additional 13 dogs participated in a 5 min long training session with the same experimenter that did not include training for initiating eye contact. After a short ball play session with the experimenter (the ball was rolled across the floor three times and the dog was encouraged to retrieve it), the dog was trained initially to touch the ball held in the experimenter's hand with its paw and once successful, to touch the ball with the paw when the ball was on the ground. First, the ball was removed, and then the experimenter kneeled on the floor in front of the dog, gained the dog's attention, and asked the dog to ‘shake’ paws with her using a verbal command and hand signal (presentation of the palm of the hand in front of the dog). When the dog touched the experimenter's hand with its paw, she clicked the clicker and the dog received a small piece of sausage as a reward. Once the dog successfully completed six clicks, the experimenter hid the ball in her hand, gave the ‘shake’ command and at the last instant turned the ball face up, and clicked and rewarded the dog for touching the ball with its paw. At all times the experimenter ensured that the dog was paying attention by calling the dog's name and gaining eye contact with the dog, before giving the verbal command and hand signal. When the dog responded correctly on a further six occasions, the experimenter placed the ball on the floor and encouraged the dog to touch it with its paw. If the dog did not respond to the command, or attempted to take the ball in the mouth, the experimenter went back to the previous successful step. This training lasted for a total of 5 min.

#### Phase 3

Immediately after being trained by the experimenter, dogs were tested in a second session of gaze following. Methods were exactly the same as in session 1, except that for the eye group, sausage was no longer needed to centre the dog in a sitting position in front of the experimenter, and the command ‘watch’ was no longer necessary, as dogs were highly motivated to attend to the experimenter after the clicker training for initiating eye contact. Again two test and two control trials were performed, which amounted to a total of four test and four control trials per dog over the two sessions.

### Data Collection and Statistical Analysis

Four digital video cameras connected to a video-recording station outside the test room were used to videotape the tests. The video-coding software Solomon Coder beta 12.09.04 (http://solomoncoder.com) was utilized to analyse the videos with a continuous sampling technique. All statistical analyses were performed in R 3.0.1 (R Core Team, 2013).

#### Clicker training for initiating eye contact

Throughout clicker training for initiating eye contact we measured the latencies until the dogs gained eye contact with the experimenter in order to investigate whether dogs differed by age in their ability to establish eye contact. The methods and results from this experiment have been reported elsewhere (Wallis et al., 2014). Since we previously showed that performance peaked in age group 4, we decided to take this group as a baseline to compare with the other age groups. Other than a short summary of the dogs' performance in this test, there is no overlap between the data sets utilized in this paper and in Wallis et al. (2014).

#### Gaze following

We measured whether or not the dog's first detectable head turn was towards the door within 2 s of the experimenter cue (first look door: yes/no). In line with previous studies analysing gaze patterns (Miklósi, Polgárdi, Topál, & Csányi, 2000; Range & Virányi, 2011; Russell, Bard, & Adamson, 1997), gaze-following ability was determined at the group level by the presence of a significant difference between the probability of looking at the door first within 2 s of the cue, in the test and the control trials.

#### Percentage time gaze experimenter face

We also measured the percentage of time the dog gazed at the experimenter's face in each of the four 10 s trials (in test and control).

#### Interobserver reliability

A randomly chosen set of 20 dogs was double coded independently by two coders, and interobserver reliability was good for percentage gaze experimenter face (*r* > 0.73, *P* < 0.001) and excellent for first look door (Cohen's Kappa = 0.91, *P* < 0.001).

#### Statistical models

We analysed the results using generalized mixed models (GLMMs, Pinheiro & Bates 2000) with a binary response term for first look door and linear mixed-effects models (LME, Davidian & Giltinan, 2003) for percentage gaze experimenter face, which was square-root transformed in order to obtain a normal distribution. Condition (test versus control), session (before versus after training), age (continuous), experiment order (test first versus control first) and direction of the cue given (left versus right) were included as fixed effects and dog identity was included as a random factor in the models. Additionally, the potentially confounding variables clicker experience, sex, neuter status and training score were included as fixed effects. Statistical models were calculated first for age as a continuous variable; we tested for linear and/or quadratic relationships. If an age effect was found, separate models were calculated with age as a categorical variable to look for specific differences between age groups. We included the two-way interactions between (1) condition and age and condition and training score to test for any age or training effects that may be restricted to one condition, and (2) session and age and session and training score to test for the effect of short-term training. Additionally, we examined the two-way interaction between group (Ball or Eye) and session, to determine whether first look door and percentage gaze experimenter face differed between the groups after training.

Normality and homoscedasticity were assessed via residual distribution plots. The terms in the models were tested using likelihood ratio tests, comparing the model containing the new term with a model excluding the new term. Nonsignificant terms (*P* > 0.05) were removed stepwise from the models. Results are presented as mean ± SD unless otherwise indicated.

## Results

### Clicker Training for Initiating Eye Contact

The results of the clicker training for initiating eye contact have been reported previously (Wallis et al., 2014). All age groups were able to improve their initial performance in latency to eye contact over the first 20 trials within the 5 min period. Group averages in trial 20 ranged from 2 to 4.2 s, compared to 4.5–8 s in trial 1. Therefore this task was effective in training the dogs to gain eye contact with the experimenter.

### First Look Door

**Figure 2:**
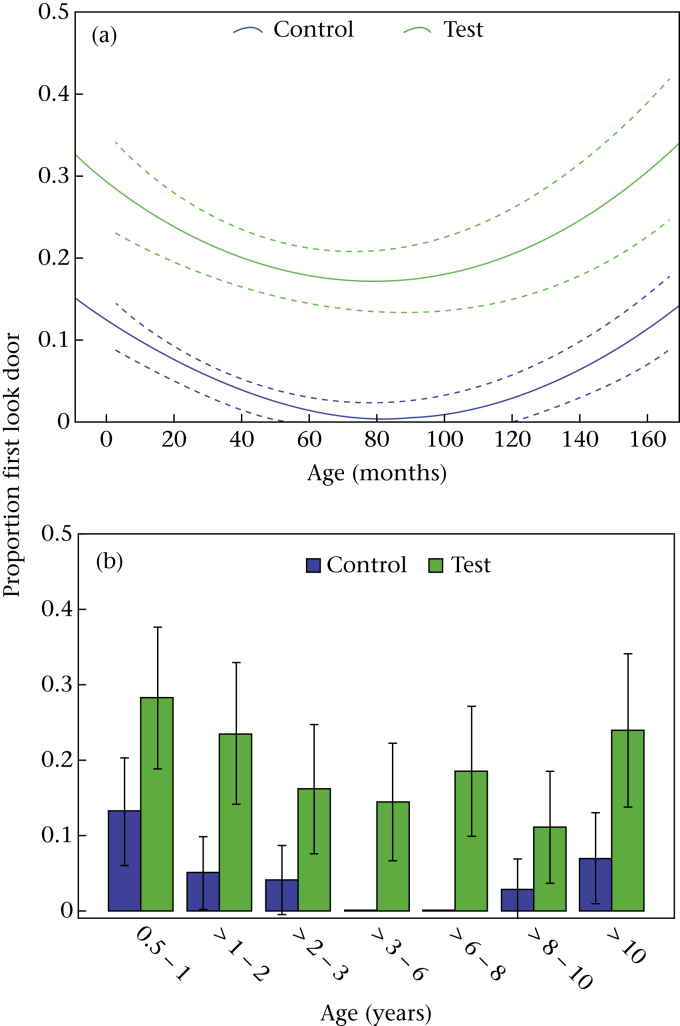
(a) Relationship between age in months and the mean proportion of dogs that first looked to the door within 2 s in the test and control conditions (with 95% confidence intervals; dotted lines). (b) The mean proportion of dogs in each age group that first looked to the door within 2 s in the test and control conditions (error bars represent SEs).

The proportion of dogs that first looked towards the door within 2 s was significantly higher in the test condition (the experimenter looked to the door) than in the control condition (the experimenter looked at her feet; Table 2, Fig. 2), providing evidence for a propensity to follow the gaze of the experimenter. Overall, 48% of the sample followed the gaze of the experimenter to the door (first look within 2 s) in at least one of the four test trials, but did not look towards the door in the control. The relationship between age and first look door was best described by a quadratic function (Table 2, Fig. 2a). We did not find any significant interactions. Dogs looked significantly more to the door in session 1 (before training) than in session 2 (after training).

When comparing the age groups, we found a significant difference in the propensity of dogs to look to the door (χ^2^ = 16.928, *P* = 0.009; Fig. 2b). Age group 1 differed significantly from the baseline (age group 4; *z* = 3.309, *P* = 0.001). That is, dogs in late puppyhood looked significantly more often towards the door within 2 s in both conditions than middle-aged dogs.

**Figure 3:**
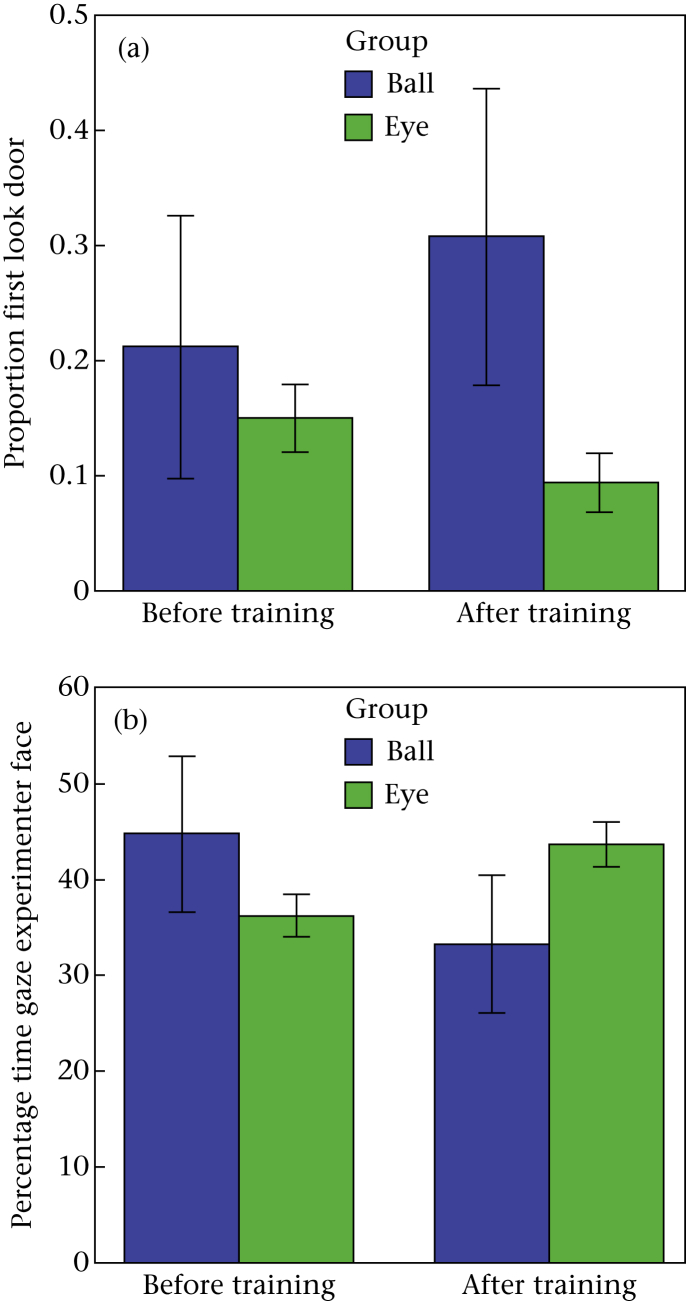
Results from Group Ball and Group Eye before and after training in (a) the mean proportion of dogs that first looked to the door within 2 s and (b) the mean percentage of time dogs gazed at the experimenter's face (error bars represent SEs).

When comparing the two groups with or without training for initiating eye contact with the experimenter, we found that dogs in Group Eye looked significantly less often to the door within 2 s than dogs in Group Ball (estimate = −1.996, χ^2^ = 12.538, *P* < 0.001). There was a significant interaction between group and session (estimate = 1.279, χ^2^ = 5.495, *P* = 0.019; Fig. 3a): the number of looks to the door increased in Group Ball after training, but decreased in Group Eye.

**Figure 4:**
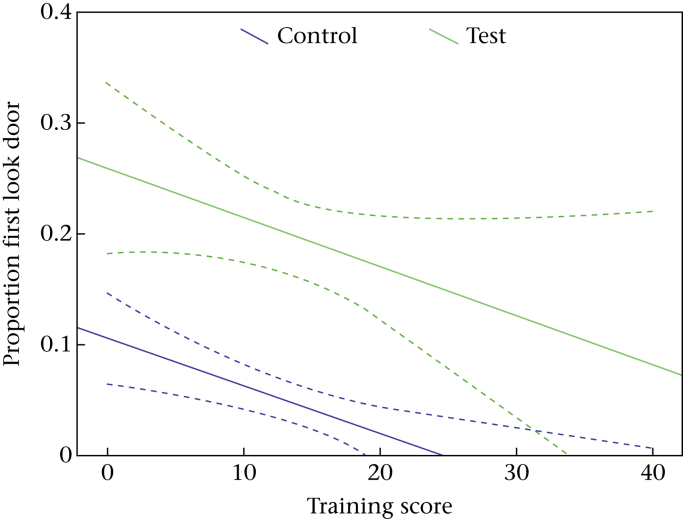
Long-term formal training-related changes in the proportion of dogs that first looked at the door within 2 s in test and control conditions (with 95% confidence intervals; dotted lines).

In a separate model, the effect of lifelong training score was examined. A significant negative linear relationship between the training score and the proportion of the first look towards the door was found (estimate = −0.05, χ^2^ = 6.198, *P* = 0.013; Fig. 4). In both conditions, dogs with more formal training experience looked significantly less to the door than dogs with little or no training experience.

### Time Spent Gazing at Experimenter's Face

**Figure 5:**
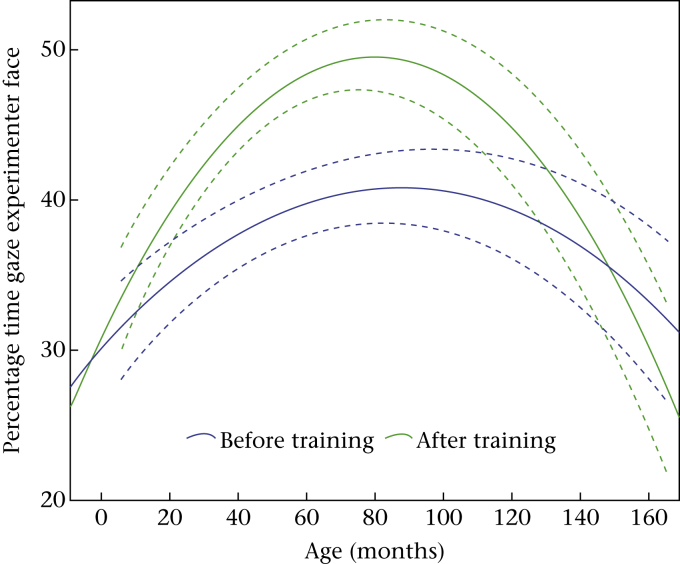
Age-related changes in the percentage of time the dogs gazed at the experimenter's face before and after clicker training for initiating eye contact (with 95% confidence intervals; dotted lines).

On average the dogs gazed at the experimenter's face for 39.7 ± 26.9% over all trials (or around 4 s per 10 s trial). There was no significant difference between percentage gaze experimenter face in the test condition and the control. The relationship between age and percentage gaze experimenter face was best described by a quadratic function (Table 3). Dogs looked for significantly less time at the experimenter's face in session 1 (36.20 ± 26.56%; before eye contact training) than in session 2 (43.62 ± 26.76%; after training; Table 3). There was a significant interaction between session and age. Percentage gaze experimenter face increased after training particularly in middle-aged dogs (Fig. 5).

When comparing the age groups, we found a significant difference in the tendency to gaze at the experimenter's face (LME: *F*_6,138_ = 2.663, *P* = 0.018). Percentage gaze experimenter face was significantly lower in age groups 1, 2 and 3 than in age group 4 (*t* > 2.52, *P* = 0.013).

When comparing Group Eye with Group Ball, we found a significant interaction between group and session (LME: *F*_1,1043_ = 17.733, *P* < 0.001; Fig. 3b). The percentage of time the dogs gazed at the experimenter's face increased after training in the eye group, but decreased in the ball group.

**Figure 6:**
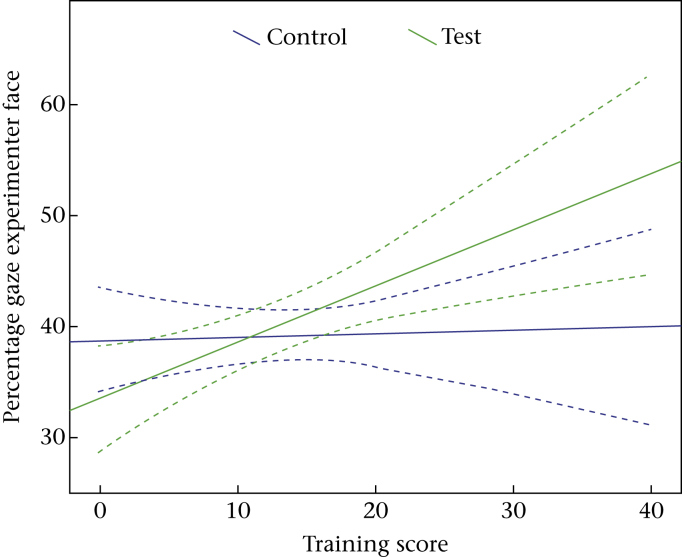
Age-related changes in the average percentage of time the dogs gazed at the experimenter's face in the test and control conditions (with 95% confidence intervals; dotted lines).

In a separate model, the effect of lifelong training score was examined. A significant interaction between condition and training score in the percentage gaze experimenter face was found (LME: *F*_1,956_ = 5.297, *P* = 0.021; Fig. 6). Dogs with more formal training experience looked significantly longer at the experimenter's face than dogs with less training experience, but only in the test condition.

## Discussion

The aims of the current study were to examine whether domestic dogs follow human gaze into distant space, and if so, whether their performance changes over their lives due to the effects of either long-term habituation or long-term learning to pay attention to humans, and finally, to determine the effects of short-term training for initiating eye contact and long-term formal training on gaze-following behaviour. Taken together, our results provide the first evidence that the domestic dog is able to follow the gaze of a human into distant space using the traditional test paradigm utilized for human infants (Scaife & Bruner, 1975), and emphasizes the effects of both lifelong formal training as well as short-term training for initiating eye contact on the propensity of dogs to follow gaze.

Our results confirm that border collie dogs show gaze-following behaviour at least when a communicatively relevant pattern of ostensive and referential signals is presented, and, additionally, that they do so outside an object choice context. All age groups were able to follow human gaze, and the propensity to follow gaze did not differ between groups. Around 50% of dogs followed gaze in at least one of the four test trials but did not look towards the door in the control. However, when all test trials were considered dogs followed gaze within 2 s in only 20% of trials. But when we removed the 2 s restriction and examined whether dogs followed gaze within 10 s, this figure jumped to 40%. Previous studies on gaze following into distant space in other species have described similar gaze-following rates between 37% and 80% (Bräuer et al., 2005; Kaminski et al., 2005; Kehmeier et al., 2011; Met et al., 2014; Range & Virányi, 2011).

Our results indicate that age (including lifelong habituation to gaze cues from humans or learning to attend to relevant human-given cues) had no effect on the gaze-following rates of dogs. However, the frequency of looks to the door showed a quadratic developmental trajectory over the dogs' lifetime, with dogs in late puppyhood and geriatric dogs showing the greatest tendency to look to the door in both test and control trials, and middle-aged dogs the lowest. As the peaks were reflected in both test and control conditions, the actual gaze-following ability of the dogs did not change over their lifetime. One explanation for the differences in the age groups could be that the youngest and oldest dogs were unable to inhibit following the salient head turn of the experimenter, and displayed greater distractibility in general, which resulted in an increased frequency of gazing to the door in the control trials and less time gazing at the experimenter's face over the 10 s trials in both conditions. There is evidence that younger and older dogs are less able to inhibit their behaviour in multiple contexts, although the reasons for decreased inhibition may be different at the different ages (Bray, MacLean, & Hare, 2014; Tapp et al., 2003). In the youngest dogs' case, this could, for instance, be due to greater general activity levels, and a higher sensitivity to external environmental stimuli (see Wallis et al., 2014). The higher distractibility of young and old dogs may have masked any potential effects of lifelong learning influences on gaze following in dogs. Perhaps for this reason, we found no evidence that during their lifelong interactions with humans, dogs would learn to pay attention to them and learn when and which of their visual cues are relevant for them.

Despite the age effects on the gazing pattern of dogs described above, across the entire sample, dogs with more formal training experience looked significantly less often to the door irrespective of condition, and in the test trials looked significantly longer into the experimenter's face than dogs with little or no training. These results provide additional evidence that dogs' human-directed behaviours are significantly influenced by their individual training experiences (Marshall-Pescini, Passalacqua, Barnard, Valsecchi, & Prato-Previde, 2009). Prior to the start of our study, the subjects had undergone several different types of formal training over their lives, all of which involved paying attention to humans and receiving subsequent verbal and visual signals from them, which seems to have inhibited their automatic gaze-following response in a social context. Since the different types of formal training the dogs engaged in may have contributed differentially to their gaze-following performance (e.g. by training them on sustained attention to humans or on anticipating a set of signals), future studies should aim to disentangle these effects by examining each training type individually (Scandurra, Prato-Previde, Valsecchi, Aria, & D'Aniello, 2015).

Surprisingly, after just 5 min of clicker training for initiating eye contact, dogs of all ages were less likely to follow gaze and spent more time watching the experimenter's face (in test and control trials). Even though the Group Eye dogs were trained to only briefly orient to the experimenter's face, in the subsequent gaze-following trials the dogs sustained their gaze to the face and ignored the gaze cue. Short-term training was most effective in dogs in early to late adulthood. Importantly, we did not find the same effect of short-term training in a second group, Group Ball, which was trained to touch a ball with their paw. In the absence of training for initiating eye contact with the experimenter, the number of looks to the door increased from session 1 to session 2, indicating that the decrease in gaze following in dogs trained for eye contact cannot be explained by a fatigue effect, or by a short-term habituation to the gaze cue. One explanation for the difference in performance between the groups is that dogs in Group Eye might have perceived the clicker training for initiating eye contact and the gaze following after the training as the same training situation, and as such, they might have simply been waiting for the experimenter to click and reward them. However, the training the dogs received in Group Ball might have been sufficiently different from the gaze-following set-up, in that the dogs did not anticipate a click or reward in the gaze-following trials, and therefore were more likely to follow the experimenter's gaze cue.

In sum, our findings do not support the hypothesis that training to pay attention to humans (either during lifelong experiences with them or during formal training) would increase the propensity of dogs to follow gaze. On the contrary, both lifelong formal training and our short experimental training for initiating eye contact created a strong tendency for dogs to sustain their gaze to the human face, and thus prevented them from following the experimenter's gaze to the door. The most likely explanation for this is that training in general creates a competing tendency to fixate on the face, which interferes with the dog's response to the referential cue given by the experimenter. It is possible that the dogs' expectation of certain verbal commands and visual signals specific for the context of their training (such as waiting for the click and treat in the clicker training for initiating eye contact) explains why they did not respond to another cue, the referential gaze of the experimenter.

There are multiple possibilities that could explain why gaze following to distant space was present in this study, but was absent in the Agnetta et al. (2000) study. Positive results found in this study may be due to the motivational effect of positive training exercises the dogs participated in with the experimenter. On at least two preceding visits, the dogs in Group Eye in our study received high-value food rewards (sausage) from the experimenter in training contexts, which is known to increase attention to humans in domestic dogs (Lindsay, 2001).

In light of the recent results from Téglás et al. (2012), who found that communicative context influenced dogs' gaze-following rates, perhaps the absence of sufficient ostensive cuing (for example addressing the individual by name) caused the dogs in Agnetta et al.’s study to ignore the actions of the experimenter. Cue saliency could also have affected dogs' performance in the current study. Ostensive cues directed towards the dog just before giving the gaze cue may have increased the saliency of the cue, and helped to maintain the dog's attention on the face long enough for it to perceive the cue direction. The fact that we used border collies as our test subjects might have influenced our results. Border collies have been selectively bred for generations as a herding dog to work cooperatively with humans, and as a consequence are particularly sensitive to human visual and acoustic stimuli (Gácsi, McGreevy, Kara, & Miklósi, 2009; McConnell & Baylis, 2010; Passalacqua et al., 2011).

Finally, studies in humans confirm that gaze following occurs more often when the other individual's gaze is oriented towards an object that is of particular relevance to the observer (Ricciardelli et al., 2013). Doors may hold particular social relevance to dogs, as even dogs as young as 6 months already have ample experience with doors, and the possibility that an individual may enter at any time. Gaze cues towards areas of particular relevance for dogs, such as the door in this case, might have facilitated the gaze-following response by providing contextual relevance.

### Conclusion

Our results provide the first scientific evidence that the domestic dog is able to follow the gaze of a human into distant space outside an object choice or barrier task context. Of the three hypotheses suggested as possible modulators of gaze following in dogs, long-term habituation, lifelong learning and formal training, only formal training was found to directly influence (decrease) gaze following. This effect was further confirmed by finding a similar, hindering, effect of short-term training for initiating eye contact on the propensity to follow gaze.

Although we found no age effect on gaze following in dogs, developmental effects on distractibility might have influenced the dogs' response. Future studies should aim to test dogs younger than 6 months, in order to more closely study the ontogeny of gaze following. An experimental investigation of long-term and short-term habituation to human gaze cues would provide essential developmental information.

In the current study, an extensive history of formal training as well as short-term training for initiating eye contact decreased the dogs' tendency to follow gaze and increased dogs' duration of gaze to the experimenter's face. We conclude that in dogs, following human gaze to distant space is modulated by training in different contexts. Our results may explain why previous studies on dogs have failed to find a gaze-following response when cues to distant space have been used, and also why dogs perform relatively poorly in comparison to other species in this task.

## Figures and Tables

**Table 1:** Age and sex of subjects

Age group	Life stage	Age (years)	Male	Female	Total
Group 1	Late puppyhood	0.5 to 1	10	13	23
Group 2	Adolescence	>1–2	10	13	23
Group 3	Early adulthood	>2–3	9	10	19
Group 4	Middle age	>3–6	9	12	21
Group 5	Late adulthood	>6–8	13	8	21
Group 6	Senior	>8–10	10	9	19
Group 7	Geriatric	>10	8	11	19
Total			69	76	145

**Table 2:** Factors affecting whether the dogs first look within 2 s was to the door

Fixed effects	Estimate	SE	Wald χ^2^	*P*
Condition: test	1.914	0.262	74.412	<0.001
Age in months: linear	−7.271	4.094	2.861	0.091
Age in months: quadratic	13.361	4.071	10.339	<0.001
Session: session1	0.708	0.214	11.560	<0.001

**Table 3:** Factors affecting dogs' mean percentage of duration of gaze to the experimenter's face over the eight trials (four control trials and four test trials) each of 10 s duration

Model term	Value	SE	*F*	*P*
Session: before training	−0.674	0.123	30.782	<0.001
Age in months: quadratic	−16.096	4.477	4.490	0.011
Session: age in months: quadratic	11.283	4.155	3.753	0.024
