# Supplementary figures and images for: Training for eye contact modulates gaze following in dogs
Source: Anim Behav. 2015 Aug;106:27–35. doi: 10.1016/j.anbehav.2015.04.020 (PMC4523690; doi:10.1016/j.anbehav.2015.04.020)

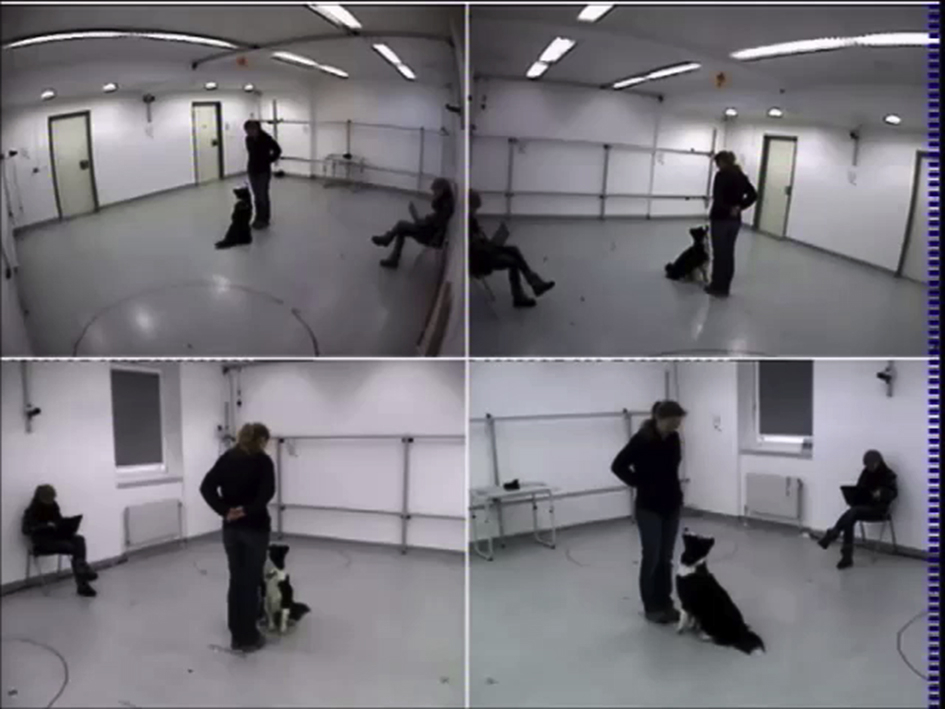

Supplement: Supplementary file 1 [file mmc1.jpg]
